# Efficacy of platelet-rich plasma in meniscal repair surgery: a systematic review of randomized controlled trials

**DOI:** 10.1186/s10195-024-00799-7

**Published:** 2024-12-18

**Authors:** Giovanni Sergio Utrilla, Irene Roman Degano, Riccardo D’Ambrosi

**Affiliations:** 1 Department of Medicine, Faculty of Medicine, University of Vic-Central University of Catalonia, Vic, Spain; 2 IRCCS Ospedale Galeazzi – Sant’Ambrogio, Milan, Italy; 3 Dipartimento di Scienze Biomediche per la Salute, Università degli Studi di Milano, Milan, Italy

**Keywords:** Meniscus repair, Platelet-rich plasma, PRP, Arthroscopy, Sports medicine, Placebo

## Abstract

**Purpose:**

This study’s primary objective was to evaluate the effectiveness of platelet-rich plasma (PRP) administration for meniscal injuries treated with meniscal repair procedures (sutures), using radiologic measures and clinical scales. The secondary objective was to identify potential bias-inducing elements in the analyzed studies.

**Methods:**

In December 2023, a systematic search was conducted in PubMed, Cochrane, Embase, and Scopus for randomized controlled trials. This review compares PRP with placebo. Three studies were finally selected. The risk of bias was assessed using Cochrane's Risk of Bias Tool 2. Radiologic evaluation of meniscal healing was measured with magnetic resonance imaging (MRI) and arthroscopic studies, while clinical evaluation was performed using four scales [Knee Injury and Osteoarthritis Outcome Score (KOOS), visual analog scale (VAS), International Knee Documentation Committee Subjective Knee Form (IKDC), and Western Ontario and McMaster Universities Index (WOMAC)] and by recording the incidence of complications.

**Results:**

The three selected studies included 139 patients; of these, 76 (54.7%) were randomly assigned to the intervention group (PRP injection) and 63 (45.3%) to the control group (placebo). The mean age of the intervention group was 37.4 ± 7.5 years, while the mean age of the control group was 36.5 ± 9.2 years. There were 41 female patients (29.5%). The median follow-up duration was 27.58 ± 17.3 months. MRI evaluation did not show a significant improvement in the PRP group in any of the studies (*p*-value = 0.41–0.54). However, when assessed by the cumulative evaluation of MRI and arthroscopy, the cumulative failure rate was significantly better in the PRP group (*p*-value = 0.04–0.048). One study that evaluated isolated arthroscopy also showed significant improvement in the PRP group (*p* = 0.003). Regarding the VAS scale, no study demonstrated a significant difference, except for one study that showed significant improvement after 6 months and in the difference between the 3rd and 6th months. The KOOS scale yielded conflicting results; one study showed no significant difference, while the other two indicated significant improvement. The IKDC and WOMAC scales were evaluated in two studies, showing opposite results. All included studies reported no complications, and one study indicated no increased risk in the treatment group.

**Conclusions:**

The results of this review indicate the necessity for further studies to make a definitive statement about the effectiveness of PRP administration in meniscal repair processes.

*Level of evidence* Systematic review and meta-analysis of articles of level 1.

**Supplementary Information:**

The online version contains supplementary material available at 10.1186/s10195-024-00799-7.

## Introduction

The menisci play a crucial role in distributing load within the knee joint. They also provide stability and lubrication to the knee joint [1–3]. Numerous studies have demonstrated that excising meniscal tissue can result in development of osteoarthritis over time. However, it has been shown that the progression of posttraumatic osteoarthritis can be slowed through meniscus refixation [4, 5]. Therefore, the objective of meniscus surgery is to conserve as much tissue as possible [5]. Meniscal repair involves various approaches, including open and arthroscopic procedures, inside-out sutures, outside-in sutures, and all-inside sutures [5]. Several factors impact the outcome of meniscal repair, including joint stability, concurrent anterior cruciate ligament (ACL) restoration, age, tear morphology, and recovery-promoting techniques [6–8]. Multiple systematic reviews and metaanalyses indicate favorable outcomes in the short and medium term following these suturing methods, with rerupture rates ranging from 10% to 19% [9]. Over the past few decades, researchers have explored various methods to enhance the effectiveness of meniscal repair, particularly through biologic augmentation. Preclinical research has shown that several biologic augmentation strategies can enhance meniscal cell activity and facilitate restoration of the meniscal tissue [10]. Specifically, platelet-rich plasma (PRP) has been utilized to exploit its anabolic capacity by releasing growth factors and bioactive compounds, resulting in improved outcomes in terms of cell proliferation and matrix synthesis [10, 11]. PRP is derived from a person’s blood and contains a high concentration of platelets, along with growth factors and other active substances [10, 11]. It has been shown to benefit tissue healing by enhancing proliferation, cell migration, angiogenesis, and extracellular matrix synthesis in various cell types in both laboratory and live models [1, 5, 9, 10]. Although clinical evidence supporting the use of PRP therapy is limited, it has been widely used for treating various musculoskeletal injuries affecting tendons, ligaments, cartilage, and bones owing to its potential benefits [9, 10]. Several growth factors, such as platelet-derived growth factor and transforming growth factor beta, have been shown to influence the inflammatory process and control chondrocyte survival, thereby maintaining tissues and repairing the meniscus [10–12]. Additionally, multiple clinical trials have confirmed that PRP injection yields favorable functional ratings and radiographic enhancement in patients with symptomatic meniscal lesions [13–16]. In contrast, several retrospective comparative studies have demonstrated that PRP administration in meniscus repair does not significantly increase pain alleviation or functional improvement [13, 17, 18]. The literature supports the notion that combining meniscal repair with ACL reconstruction (ACLR) leads to improved healing, likely owing to the release of mesenchymal stem cells (MSCs) from the tibial tunnel. This further confirms the advantages of using biologic factors to enhance meniscal healing. While biologic augmentation approaches have been implemented in clinical practice, their actual effectiveness remains a topic of debate [16, 18, 19].

The aim of this systematic review was to evaluate the functional and radiologic effects of PRP compared with placebo in meniscus repair. The hypothesis was that PRP would improve functional and radiologic outcomes in meniscal repair.

## Methods

This systematic review followed the Preferred Reporting Items for Systematic Reviews and Meta-Analyses (PRISMA) guidelines and was registered in the PROSPERO registry (registration no. CRD42024535723) [20, 21].

The PICO questions of this study are represented in Supplementary Table 9 [22].

### Eligibility criteria

We included randomized clinical trials (phase 1 or 2) published in English, Italian, or Spanish. Studies had to involve patients aged 18 years or older, treated arthroscopically for isolated meniscal lesions (either medial or lateral), with meniscal repair and with or without PRP augmentation, and evaluated for clinical and radiological outcomes, failure rates, and safety.

Studies were excluded if they involved animals, patients younger than 18 years, follow-up shorter than 6 months, or patients with discoid meniscus, previous inflammatory diseases, or Kellgren–Lawrence scale > 2 before surgery. We also excluded articles in which patients received PRP injections combined with other drugs/compounds or when other surgical procedures, such as open procedures or concomitant ACL reconstruction, were performed.

### Outcome measures

The outcome measures extracted from the studies included four scales: International Knee Documentation Committee Subjective Knee Form (IKDC), Knee Injury and Osteoarthritis Outcome Score (KOOS), visual analog scale (VAS), and Western Ontario and McMaster Universities Index (WOMAC) [23]. We also extracted information on meniscal healing via magnetic resonance imaging (MRI) or arthroscopy, the safety of the process, and failure rates.

The primary outcome was the score on the KOOS and VAS scales. Secondary outcomes included the IKDC and WOMAC scores, as well as assessments of meniscal healing, failure rates, and safety.

The KOOS scale is a 41-item questionnaire that assesses short- and long-term outcomes following a knee injury. It is divided into five domains: pain (9 items), symptoms (7 items), activities of daily living (17 items), ability to perform sports activities (5 items), and knee-related quality of life (4 items) [6]. The raw score for each domain was calculated as previously explained [23].

The VAS evaluates the intensity of a symptom, particularly the subjective sensation of pain. The tool is applied by asking the patient to rate their pain/sensation from 0 to 10, where 0 indicates absent sensation and 10 indicates the highest intensity possible [23].

The IKDC scale assesses symptoms and function in patients with knee disorders. It includes 11 items (7 related to knee symptoms, 2 to function, and 2 to sports activities) and is evaluated over a total of 100 points (highest level of function and lowest of symptoms) [23]. The final raw score is calculated using the formula: (sum of all items/87) × 100 [9].

The WOMAC is a 24-item questionnaire used to evaluate the severity of knee/hip osteoarthritis symptoms [23]. It has three subscales: pain (5 items, with a score from 0 to 20), stiffness (2 items, with a score from 0 to 8), and physical function (17 items); its overall score ranges from 0 to 68 [23].

Meniscal healing was assessed via MRI and arthroscopic studies as follows: complete healing was defined as full meniscus integrity, partial healing as at least 50% tear healing, and healing failure as no visible healing or healing < 50% [24].

Safety was evaluated through the analysis of perioperative and postoperative complications (local infections, blood clots, nerve damage, pain around the injection site, and tissue damage) reported in the studies. We compared the number of complications in the PRP group with those in the control group [25].

Failure was defined as [26, 27]:

- 1. No visible healing or healing lower than 50% of the tear width (by MRI/arthroscopy)
- 2. Unstable repair (by MRI/arthroscopy)
- 3. Detection of contrast media within the meniscal body in MRI studies

### Complications and adverse events

Undesirable clinical developments that were not present at baseline or which increased in severity after treatment were classified as adverse events. The duration, type, and severity of adverse events are recorded as defined in Supplementary Table 1 [25].

Injection-related complications were defined as any deviation from the normal postoperative course owing to the implants [25].

All studies considered a *p*-value < 0.05 as significant.

### Information sources and search

PubMed, Scopus, EMBASE, and Cochrane Library databases were systematically searched from January 1990 to December 2023 by two independent reviewers (G.S.U. and R.D.).

The following search strategy was employed: (((“meniscus” or “menisci” or “meniscal” or “meniscopathy” or “Meniscal”) and (“injury” or “repair” or “damage” or “tear”)) or (“Meniscal injury” or “meniscus injury” or “meniscal injury”)) and (“PRP” or “platelet-rich plasma”).

No deviations from the registered protocol were reported.

### Data collection and analysis

#### Study selection

The articles retrieved from the search were initially filtered to exclude studies that were not randomized controlled trials and duplicates. The authors then performed a first selection by title and, if deemed relevant, screened further by full-text reading. Papers that did not meet the inclusion criteria were excluded.

To minimize bias, the authors reviewed and discussed the remaining articles and the excluded ones. In case of disagreement, the senior investigator made the final decision. At the end of the process, the reference list of the included studies, as well as the reference list of similar systematic reviews, was manually searched.

#### Data collection process

The first two authors extracted the data from the selected articles, using a computerized tool created with Microsoft Access (version 2010, Microsoft Corp, Redmond, WA). For each study, we extracted the country, study design, sample size, mean age, sex, BMI, PRP preparation, PRP injection protocol, median follow-up, rate of complications, and defined outcomes.

#### Evaluation of the risk of bias of included studies

The risk of bias was evaluated using the Cochrane Risk of bias 2 (ROB2) tool [15]. This checklist evaluates five domains (risk of bias arising from the randomization process, due to deviation from intended interventions, due to missing outcome data, related to the measurement of the outcomes, and related to the selection of reported results) and allows evaluation of the overall risk of bias of the article. Risk of bias graphs were obtained using the Cochrane’s Robins tool [28, 29].

## Results

**Fig. 1:**
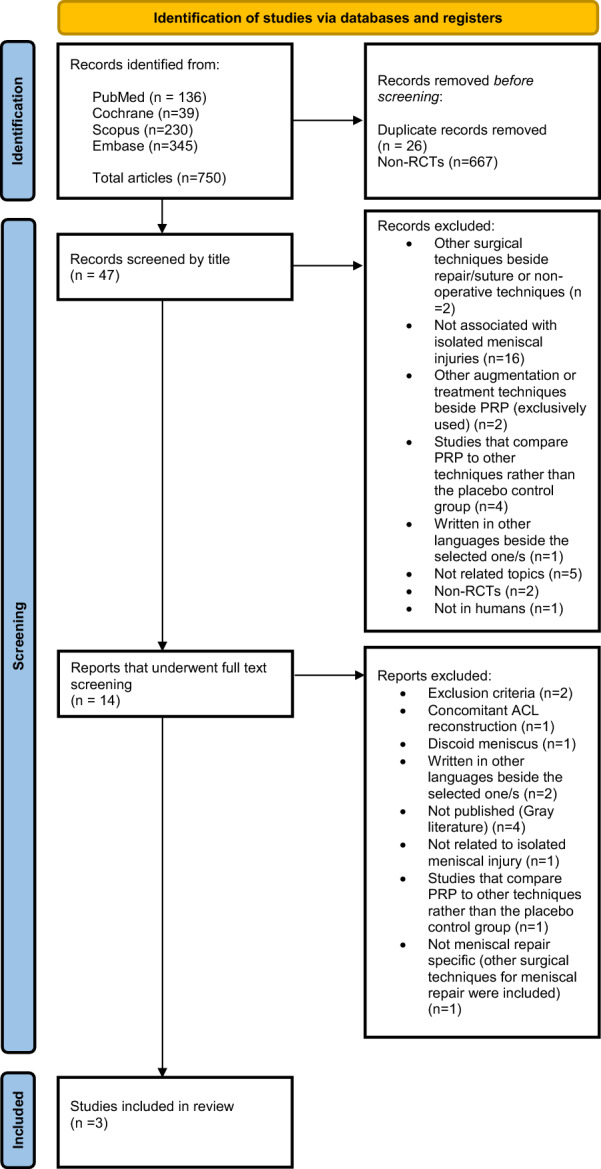
PRISMA flowchart summarizing the article selection and screening process

After the selection process and assessment of the inclusion criteria, three articles were included in the study (Fig. 1) [26, 27, 30].

**Fig. 2:**
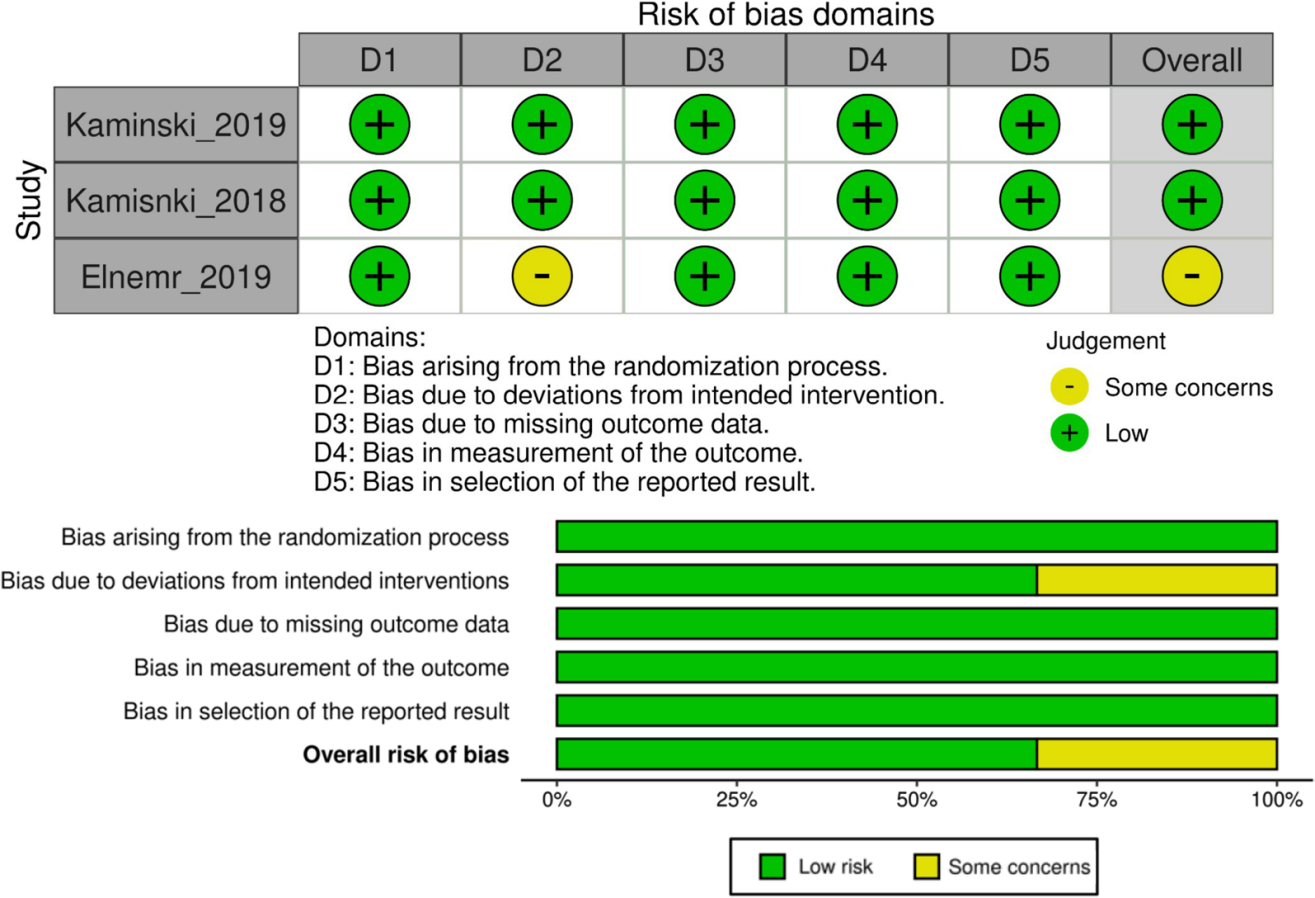
Traffic light plot and bar plot representing the risk of bias domains and overall risk of bias of the included articles evaluated through the Cochrane Risk of Bias 2 (ROB2) tool

The risk of bias assessment showed that, out of the three included studies, two had a low risk of bias, while one raised some concerns in the domain of “deviation from intended interventions” owing to a lack of information (Fig. 2).

### Patient characteristics

The three selected studies included 139 patients [26, 27, 30]. Of these, 76 (54,7%) were randomly assigned to the intervention group (PRP injection) and 63 (45.3%) to the control group (placebo, Table 1). The mean age of the intervention group was 37.4 ± 7.5 years, while that of the control group was 36.5 ± 9.2 years. The number of female patients (29.5%) was 41.

**Table 1:** Overview of included studies

Author	Year	Country	Study design	Sample size (PRP/control)	BMI (PRP/control)	Years [mean age] (PRP/control)	Gender (M/F)	PRP preparation	PRP injection protocol	Median follow-up (months)	MM:LM
Kaminski et al.	2019	Poland	Prospective, randomized, placebo-controlled, double-blind study	72 (42/30)	27/28	44/46	41/31	Activated using autologous thrombin	Minimally invasive intrameniscal application (6–8 mL)	23 months (92 weeks)	71:1
Kaminski et al.	2018	Poland	Prospective, randomized, double-blind, placebo-controlled, parallel-arm study	37 (19/18)	NR	30/26	30/7	ELISA and blood analyzer; activated using autologous thrombin	Injected into meniscal repair site (8 mL)	54 months (243 weeks)	NR
Elnemr et al.	2019	Egypt	Prospective, randomized, double-blind, placebo-controlled	30 (15/15)	27.2/25.5	27.7/30.1	27/3	Calcium-chloride activated	Immediate injection into meniscal repair site (without storage) (5 ml)	6 months (26 weeks)	14:16

*M/F* males/females, *MM:LM* medial meniscus:lateral meniscus

Two studies specified whether the injuries were associated with the medial or lateral meniscus (102 patients) [26, 30]. Of those, 85 (83.33%) patients had a medial meniscus injury, and 17 (16.67%) a lateral meniscus injury.

The median follow-up duration was 27.58 ± 17.3 months.

#### Intervention

PRP characteristics were reported in the three included studies (Table 2). In two studies, the presence of leukocytes in the PRP formulation was noted [26, 27]. Two studies injected the PRP solution with an intrarepair site injection [27, 30], while the other used ultrasound-guided meniscus trephination [26]. In one of the studies, more than one injection was performed [30]. One of the included articles [26] classified the PRP type according to the classification by Harrison et al. [31], specifying it as a Red-L-PRP IIB-1 class PRP, which is erythrocyte and leukocyte rich for the use of PRP with activators (class II) with a platelet count of 900–1700 × 10^3^ µL^–1^ (B).

**Table 2:** PRP treatment protocol

Author	PRP characteristics	PRP protocol
Kaminski et al. 2019	Red-l-PRPIIB-1 class Activated with CaCl_2_ (20 mM) Contains leukocytes	Number of injections: 1 Volume: 6–8 mL Ultrasound-guided meniscal trephination
Kaminski et al. 2018	Activated with thrombin Presence of leukocytes Validated using enzyme-linked immunosorbent assay (ELISA) and a blood analyzer	Number of injections: 1 Volume: 8 mL Intrarepair site injection
Elnemr et al. 2019	0.2 mL of 10% calcium chloride was used as activator	Number of injections: 6 Interval: 1 month Volume: 5 mL Intrarepair site injection

### Radiologic and clinical outcomes

Two studies reported radiologic outcomes [26, 27] (Supplementary Figs. 1–4). Both studies used MRI and/or arthroscopy to analyze the radiologic outcomes. When assessed through MRI, the evaluation did not show a significant improvement in the PRP group in any of the studies (*p*-value = 0.41–0.54). If assessed through cumulative evaluation of MRI and arthroscopy, the cumulative failure rate was significantly better in the PRP group (*p*-value = 0.04–0.048). One study that evaluated isolated arthroscopic studies only [27] showed significant improvement in the PRP group (*p* = 0.003).

All three studies reported clinical outcomes following treatment. All of them used VAS and KOOS scales. Two articles also utilized the WOMAC and IKDC scales [26, 27].

Regarding the VAS scale, no study showed a significant difference, except for one study that demonstrated significant improvement after 6 months and in the difference between the 3rd and 6th months [30]. The KOOS scale showed conflicting results; one study showed no significant difference (*p* > 0.05) [26], while the other two indicated significant improvement (*p* < 0.05) [27, 30]. The IKDC and WOMAC scales were evaluated in two studies, yielding opposite results [26, 27]. One study found significant improvement (*p* < 0.05) [27], while the other did not (*p* > 0.05) [26]. Detailed results are reported in Table 3.

**Table 3:** Radiologic and clinical outcomes (summarized and visually represented in Supplementary Tables 2–7)

Author	MRI evaluation	MRI and arthroscopy evaluation (cumulatively)	Arthroscopic evaluation	KOOS	VAS	IKDC	WOMAC
Kaminski et al. 2019 (for the control group versus PRP group; data are presented as mean ± standard error (CI 95%) unless otherwise indicated	Total of patients who underwent this procedure: 54 (out of 72) - PRP group (27 patients) • Healed: 10 • Partially healed: 4 • Failed: 13 - Control group (27 patients) • Healed: 5 • Partially healed: 3 • Failed: 19 (*p* = 0.41)	Total of patients who underwent this procedure: 41 (out of 72) - PRP group (25 patients) • Healed: 11 • Partially healed: 4 • Failed: 10 - Control group (26 patients) • Healed: 7 • Partially healed: 4 • Failed: 15 (*p* = 0.04)	NR	Control group: Pre-procedure - Pain 65.30 ± 0.54 (59.51–71.10) • Symptoms 69.86 ± 0.62 (63.18–76.54) • ADL 68.42 ± 0.66 (61.33–75.50) • S/R 33.50 ± 0.62 (26.84–40.16) • QoL 35.00 ± 0.49 (29.73–40.27) - Post trephination • Pain 89.00 ± 0.63 (83.19–94.81) • Symptoms 90.42 ± 0.56 (85.26–95.58) • ADL 92.38 ± 0.61 (86.80–97.95) • S/R 78.98 ± 1.10 (68.83–89.12) • QoL 68.18 ± 1.08 (58.28–78.08) PRP group: - Pre-procedure • Pain 57.48 ± 0.30 (57.18–57.78) • Symptoms 63.53 ± 0.39 (63.23–63.83) • ADL 63.70 ± 0.37 (63.40–64.00) • S/R 35.83 ± 0.51 (35.53–36.14) • QoL 37.90 ± 0.26 (37.59–38.20) - Post trephination • Pain 87.24 ± 0.36 (82.99–91.48) • Symptoms 92.03 ± 0.27 (88.80–95.26) • ADL 89.36 ± 0.36 (85.07–93.64) • S/R 69.52 ± 0.77 (60.29–78.74) • QoL 67.06 – 0.55 (60.56–73.56) [*p* value: • Pain (*p* = 0.22) • Symptoms (*p* = 0.27) • ADL (*p* = 0.25) • S/R (*p* = 0.11) • QoL (*p* = 0.42)]	Control group: -Pre procedure 4.40 ± 0.07 (3.55–5.25) -Post trephination 2.05 ± 0.08 (1.27–2.82) PRP group: -Pre procedure 5.38 ± 0.05 (4.77–5.990 -Post trephination 1.97 ± 0.05 (1.40–2.55) [(*p* value: 0.39)]	Control group: -Pre procedure 54.92 ± 0.54 (49.08–60.77) - Post trephination 88.12 ± 0.89 (79.97–96.28) PRP group: -Pre procedure 51.99 ± 0.34 (47.62–56.36) -Post trephination 85.98 ± 0.52 (79.79–92.16) [(*p* value: 0.36)]	Control group: - Pre procedure 28.93 ± 0.61 (22.42–35.45) - Post trephination 7.50 ± 0.59 (2.06–12.94) PRP group: - Pre procedure 34.36 ± 0.35 (29.90–38.82) - Post trephination 9.72 ± 0.32 (5.95–13.48) [(*p* value: 0.21)]
Kaminski et al. 2018	Total of patients who underwent this procedure: 11 (out of 37) - PRP group (6 patients) • Healed: 3 • Partially healed: 1 • Failed: 2 - Control group (5 patients) • Healed: 3 • Partially healed: 1 • Failed: 8 (*p* = 0.54)	Total of patients who underwent this procedure: 37 (out of 37) - PRP group (20 patients) • Healed: 14 • Partially healed: 3 • Failed: 3 - Control group (17 patients) • Healed: 7 • Partially healed: 1 • Failed: 9 (*p* = 0.048)	Total of patients who underwent this procedure: 26 (out of 37) - PRP group (14 patients) • Healed: 11 • Partially healed: 2 • Failed: 1 - Control group (12 patients) • Healed: 3 • Partially healed: 1 • Failed: 8 (*p* = 0.003)	Control group: - Pre-procedure • Pain 55.15 ± 1.04 (46.49–63.81) • Symptoms 44.84 ± 1.13 (35.44–54.25) • ADL 58.95 ± 1.34 (47.81–70.09) • S/R 24.44 ± 1.73 (10.03–38.86) • QoL 22.57 ± 0.91 (15.02–30.12) - Post-procedure • Pain 92.85 ± 0.43 (89.83–95.87) • Symptoms 92.33 ± 0.48 (88.94–95.73) • ADL 95.14 ± 0.38 (92.47–97.81) • S/R 77.65 ± 1.26 (68.73–86.56) • QoL 66.18 ± 1.17 (57.94–74.42) PRP group: - Pre-procedure • Pain 58.81 ± 0.83 (51.68–65.94) • Symptoms 51.88 ± 1.15 (42.08–61.68) • ADL 66.68 ± 0.95 (58.55–74.81) • S/R 25.53 ± 1.32 (14.29–36.76) • QoL 22.57 ± 0.91 (15.02–30.12) - Post-procedure • Pain 96.06 ± 0.23 (94.22–97.91) • Symptoms 96.23 ± 0.31 (93.79–98.67) • ADL 98.18 ± 0.13 (98.13–100.24) • S/R 89.44 ± 0.86 (82.68–96.21) • QoL 80.90 ± 1.09 (72.34–89.47) [P value of Control group and PRP treated group previously to the intervention: • Pain (*p* = 0.53) • Symptoms (*p* = 0.32) • ADL (*p* = 0.28) • S/R (*p* = 0.91) • QoL (*p* = 0.37) *p*-Value of control group and PRP-treated group after the intervention: • Pain (*p* = 0.035) • Symptoms (*p* = 0.029) • ADL (*p* = 0.0004) • S/R (*p* = 0.009) • QoL (*p* = 0.008)]	Control group: -Pre procedure 5.06 ± 0.13 (4–6.11) -Post trephination 0.89 ± 0.08 (0.33–1.44) PRP group: -Pre procedure 6.21 ± 0.13 (5.13–7.29) -Post trephination 0.84 ± 0.10 (0.04–1.65) [*p* value: Before the intervention (*p* = 0.14) After the intervention (*p* = 0.15)]	Control group: -Pre procedure 41.7 ± 0.84 (34.7–48.69) -Post trephination 84.77 ± 0.92 (78.24–91.29) PRP group: -Pre procedure 40.92 ± 0.92 (33.09–48.74) -Post trephination 97.56 ± 0.63 (92.62–102.49) [*p* value: Before the intervention (*p* = 0.88) After the intervention (*p* = 0.001)]	Control group: -Pre procedure 38.61 ± 1.19 (28.74 − 48.49) -Post trephination 3.95 ± 0.33 (1.58 − 6.31) PRP group: -Pre procedure 32.26 ± 0.90 (24.55–39.97) -Post trephination 0.95 ± 0.13 (−0.07 to 1.96) [*p* value: Before the intervention (*p* = 0.25) After the intervention (*p* = 0.002)]
Elnemr et al. 2019	NR	NR	NR	Control group: -Pre procedure 66.3 ± 8.7 -Post-procedure *77.1 ± 6.3{3 months] *81.1 ± 6.1 [6 months] PRP group: -Pre procedure 62 ± 9.8 -Post procedure * 83.5 ± 4.4 {3 months] * 86.2 ± 4 [6 months] % of change in PRP group: ↑42.2 ± 22.5 % of change in control group: ↑24.3 ± 18.8 [*p* value:→ Before the intervention (*p* = 0.217) 3 months after the intervention (*p* = 0.003) 6 months after intervention (*p* = 0.012) % Change between 3rd and 6th month (*p* = 0.014)]	Control group: -Pre procedure 8 (5 -8) -Post trephination * 5 (1–8) {3 months] * 5 (1–7) [6 months] PRP group: -Pre procedure 9 (7—10) -Post trephination * 4 (1–7) {3 months] * 1 (1–3) [6 months] % of change in PRP group: ↓84.9 ± 6.9 % of change in control group: ↓38.4 ± 37.4 % of change in control group: [*p* value:→ Before the intervention (*p* = 0.09) 3 months after the intervention (*p* = 0.474) 6 months after intervention (*p* = 0.003) % change between 3rd and 6th month (*p* < 0.001)]	NR	NR

Possibly elements leading to bias, for future studies (summary table)
• MRI sensibility/interpretation and the importance of second-look arthroscopic studies
• Differentiation between acute and chronic injuries (chronicity of the injuries)
• Localization of the injury (topographic differentiation)
• PRP administration technique
• PRP subtypes
• Age of the population
• Medial meniscus versus lateral meniscus

### Complications

All included studies reported no complications [26].

## Discussion

The most important findings of the current systematic review reveal that PRP in conjunction with meniscal suture remains controversial, highlighting the need for more studies that consider additional parameters to effectively evaluate PRP’s effectiveness.

The evidence indicates discordant results, suggesting that more parameters must be considered to formulate a clear statement regarding the effectiveness of this technique.

Current literature reports results comparable to ours, confirming the ambiguity in existing studies. For instance, Li et al. [10] found that VAS and failure rates significantly improved in the PRP-treated group, while IKDC and Lysholm scores (another patient-reported evaluation tool related to knee functionality) reported the opposite.

In a systematic review by Yaodong Wang et al. [32], VAS and failure rates showed significant improvement, but IKDC and Lysholm did not. The authors noted the need for uniform standards in PRP preparation and application to avoid heterogeneity among studies.

Xie et al. [33] demonstrated heterogeneity in results between evaluations of meniscal healing and outcomes reported via VAS and Lysholm.

Migliorini et al. [13] found no improvement in the PRP group across any of the evaluation tools used (failure rate, VAS, Lysholm, and IKDC). They also highlighted limitations in the data and the small size of the studies.

Finally, Xie et al., Sochacki et al., and Haunschild et al. identified significant limitations, including the need for further studies, specifically indicating a lack of suitable RCTs for analysis, a large age gap between participants in the studies [25], low study quality [26], insufficient evidence, and heterogeneity among PRP preparations [33–35].

Another bias in the current literature regarding the use of PRP, particularly in meniscal surgery, is the variability in the preparation and administration of blood derivatives [36–38].

Blood derivatives are classified differently based on preparation methods and include PRP, leukocyte-rich PRP, platelet-rich fibrin, growth factors (GF)-rich PRP, platelet-rich fibrin matrix, autologous concentrated plasma, pure PRP, platelet gel, autologous conditioned plasma, and autologous protein solution, among others. These products have varying concentrations of blood cells, plasma, or fibrinogen, resulting in different levels of growth factors and bioactive compounds. The biological characteristics of those products may differ and affect their efficacy in knee osteoarthritis. Leukocytes are currently a contentious topic, as in vitro research has shown they can promote the release of catabolic and proinflammatory chemicals. However, a recent clinical investigation did not validate an increase in inflammatory chemicals within the synovial fluid. Furthermore, the only comparative trial conducted demonstrated similar outcomes between leukocyte-rich and leukocyte-poor formulations. Recent reports have also indicated favorable results from leveraging the pleiotropic effects of leukocyte-rich concentrates, leaving the inquiry into the in vivo function of leukocytes unresolved. In this context, it is crucial to emphasize that, although the benefits of leukocyte-poor preparations have been previously proposed, such assertions are based on indirect comparisons that carry a significant risk of bias. Moreover, the precise composition of PRP is inadequately documented in much existing research, and classifications are sometimes inconsistent, conflating PRPs with normal leukocyte levels with those exhibiting total leukocyte depletion. Future trials should enhance the characterization of PRP products to elucidate the role of leukocytes. The existing evidence on this matter remains insufficient, necessitating future high-quality research that directly compares various PRP formulations to enable trustworthy analysis and definitive findings [36–38].

Apart from leukocyte content, protocols vary regarding blood volume collected, anticoagulant usage, centrifugation frequency and velocity, final volume achieved, total platelet count, their integrity and activation technique, and the option for cryopreservation or utilization of fresh products, all of which may affect the characteristics of the releasate. Furthermore, many application modalities have been documented, involving single injections or injection cycles with differing volumes and concentrations, ultimately administering diverse dosages of releasate. This may be a significant factor, as preclinical and clinical research has indicated the importance of growth factor dose [36–38].

In our systematic review, the evaluation of meniscal healing by arthroscopy or by MRI + arthroscopy showed a significantly improved PRP-treated group.

The use of MRI to evaluate meniscal healing in the literature remains debated; in 2020, Faunø et al. investigated the diagnostic accuracy of MRI and clinical assessment in determining failed meniscal repair in symptomatic meniscal repair patients, as verified by re-arthroscopy [39]. Eighty patients were included, all of whom had undergone primary meniscal repair followed by MRI and re-arthroscopy due to clinical symptoms of a meniscal lesion. A validated semiquantitative scoring system was employed to identify MRI-diagnosed healing failure. The clinical assessment was divided into joint swelling, joint-line tenderness, locking, and a positive McMurray’s test. The sensitivity, specificity, positive predictive value (PPV), and negative predictive value of MRI results showed healing in 22 (27.5%) of the menisci and 58 (72.5%) unhealed menisci, whereas second-look arthroscopy identified 15 (19%) healed menisci and 65 (81%) unhealed menisci. The isolated MRI findings yielded sensitivity, specificity, PPV, and a negative predictive value of 0.85, 0.8, 0.95, and 0.55, respectively. The PPVs of the clinical assessments were 0.78, 0.85, and 0.94, with one, two, and three clinical findings, respectively. A grade 3 MRI combined with joint-line tenderness presented a PPV of 0.98 [39].

In contrast, Yamasaki et al. compared the accuracy of MRI T2 mapping and conventional MRI in assessing meniscal healing after repair, demonstrating that MRI T2 mapping allowed differentiation of healing status after meniscal repair, with high sensitivity and specificity compared with conventional MRI [40].

To better analyze the results of the current systematic review, other important aspects must be considered.

First, acute and chronic injuries must be distinguished; in the study by Kaminski et al., the authors evaluated only degenerative meniscal lesions, while the other two studies did not specify the chronicity of the tears [26, 27, 30]. The other two studies could have included meniscal injuries of various etiologies, not only degenerative ones. These findings could suggest that the effectiveness of the PRP may be related to the chronicity of the injuries.

Van der Wal et al. in 2016 evaluated failure rates and clinical outcomes of arthroscopic meniscal repair in relation to injury chronicity [41]. A total of 238 menisci were repaired in 234 patients. ACLs were reconstructed in almost all ACL-deficient knees (130 out of 133). The time interval between injury and repair was divided into acute (< 2 weeks), subacute (> 2 to < 12 weeks), and chronic (> 12 weeks). No significant difference was found for any interval between trauma and repair. The interval between trauma and arthroscopic meniscal repair does not influence the failure rate. Differences in survival rates of meniscal repair depend more on the location of the lesion and ACL status rather than the chronicity of injury [41].

The current systematic review did not specify the localization of the injury and whether this factor plays a key role in the final rates of failures and clinical outcomes.

Cinque et al. examined outcomes after inside-out meniscal repair in all three meniscal vascularity zones [42]. The authors concluded that patients who underwent inside-out meniscal repair significantly improved in subjective outcome measures at a minimum of 2 years follow-up, regardless of the meniscal tear zone. Inside-out meniscal repair is recommended for potentially reparable meniscal tears in all three vascular zones; however, improved outcomes can be achieved when performed acutely, in the absence of full-thickness femoral condyle chondral injuries, and in the red–red and red–white zones [42].

## Limitations

This study has multiple limitations. The literature search uncovered significant heterogeneity among the studies examined in terms of the range of injuries treated, the time elapsed between injury and surgery, and variations in protocols used (such as the preparation and administration of PRP). Specifically, the participants in each study had different types of lesions and mechanisms of injury, and the presence of heterogeneity in the data may complicate the interpretation of the results.

The clinical significance of this systematic review is that PRP can be beneficial in treating meniscal repair, improving pain and functional outcomes with a low rate of complications, being demonstrated to be a safe procedure. Nevertheless, additional rigorous investigations standardizing PRP preparation and administration protocols, and including larger sample sizes, are required to verify the accuracy of the current results.

## Conclusions

There is a paucity of literature regarding RCTs of meniscal repair with adjunct PRP. The few studies included in the current assessment reported different procedures, which makes it impossible to state with certainty the effects of PRP in meniscal repair and highlights the need for further research on this topic. Despite conflicting data, most patients who underwent meniscal repair and PRP administration achieved satisfactory clinical and functional outcomes, with a low rate of postoperative complications.

## Supplementary Information

Additional file 1.

## Data Availability

Raw data are available upon request to the corresponding author.
