# Supplementary material for: Efficacy of platelet-rich plasma in meniscal repair surgery: a systematic review of randomized controlled trials
Source: J Orthop Traumatol. 2024 Dec 18;25:63. doi: 10.1186/s10195-024-00799-7 (PMC11656006; doi:10.1186/s10195-024-00799-7)

# Appendix

***Supplementary table 1:*** Definition of adverse events with their severity and eventual required intervention used to evaluate and classify the possible complications presents in the included studies.


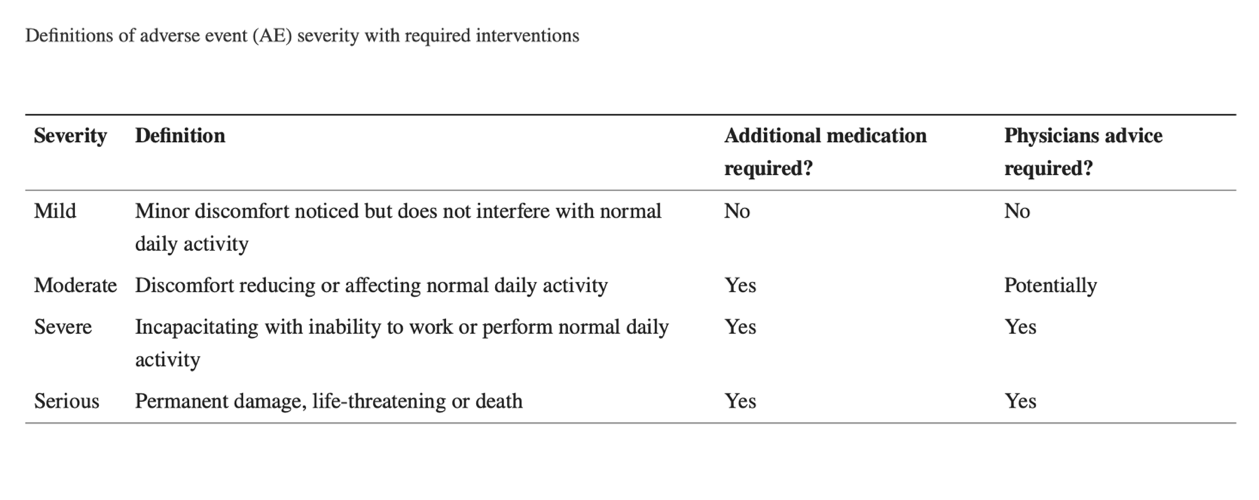


***Supplementary figure 2:*** Graphic representation of the prevalence of the different meniscal healing grades (previously explained in the methods) evaluated trough MRI in Kaminsky et al. 2019 [13].


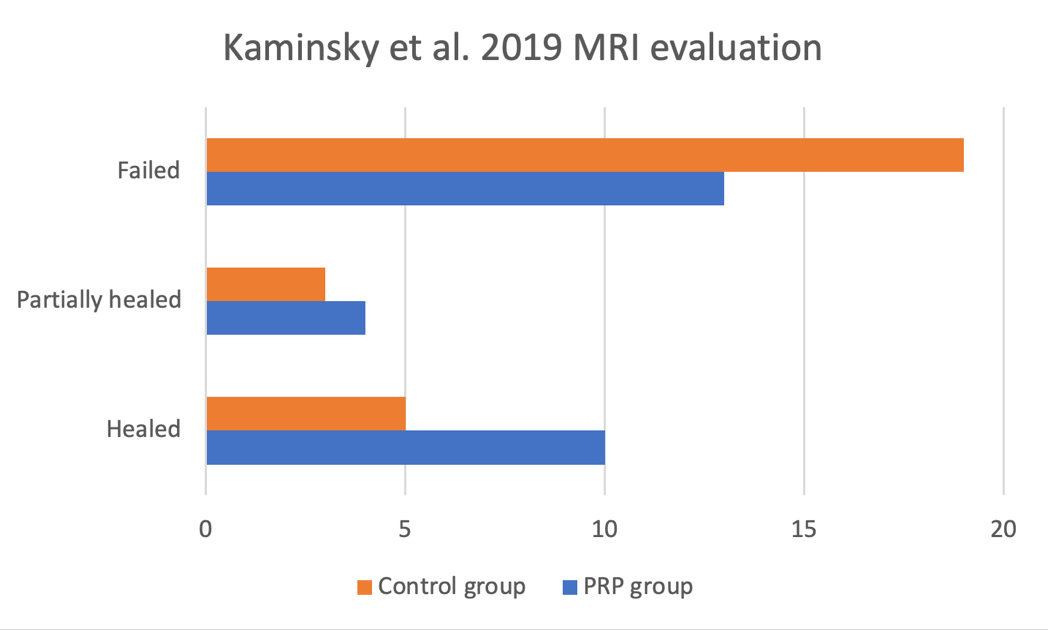


***Supplementary figure 3:*** Graphic representation of the prevalence of the different meniscal healing grades (previously explained in the methods) evaluated cumulatively via MRI + Arthroscopic studies in Kaminsky et al. 2019 [13].


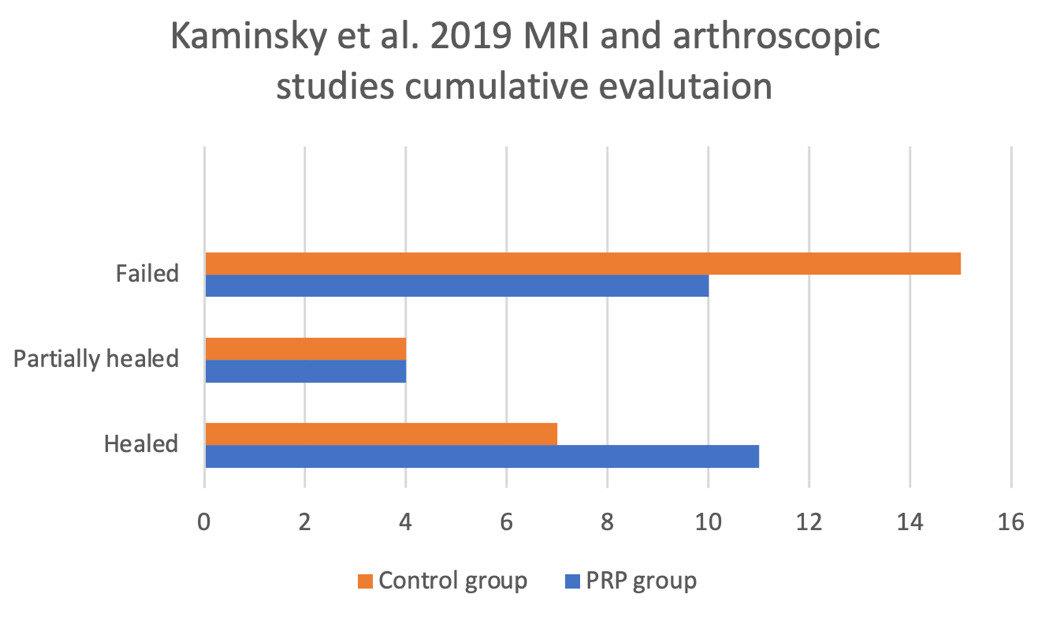


***Supplementary figure 4:*** Graphic representation of the prevalence of the different meniscal healing grades (previously explained in methods) evaluated trough MRI in Kaminsky et al. 2018 [14].


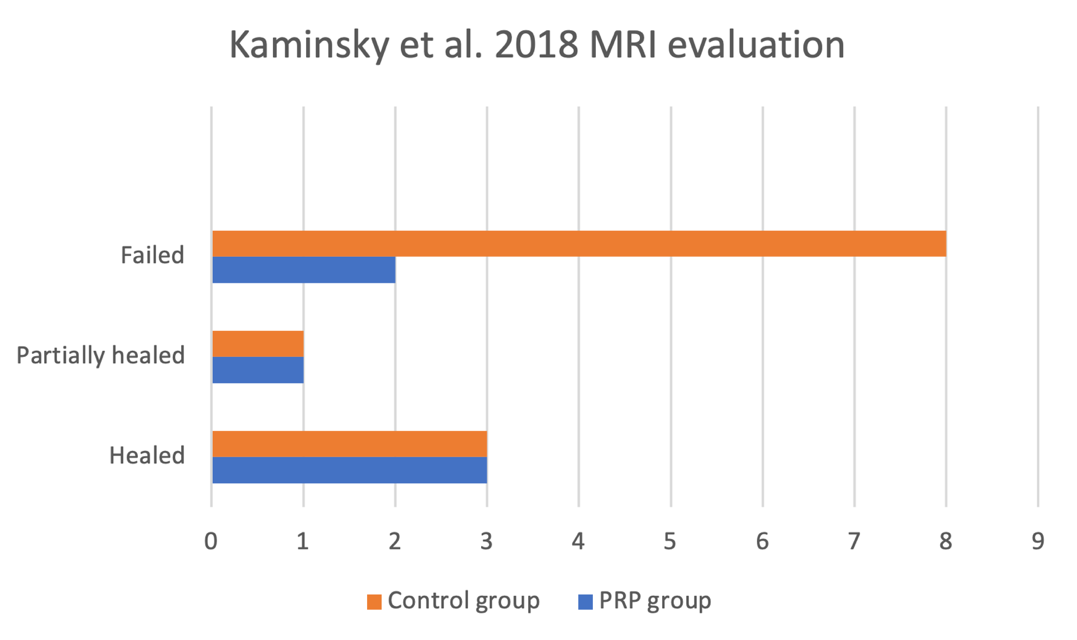


***Supplementary figure 5:*** Graphic representation of the prevalence of the different meniscal healing grades (previously explained in methods) evaluated trough arthroscopic studies in Kaminsky et al. 2018 [14].


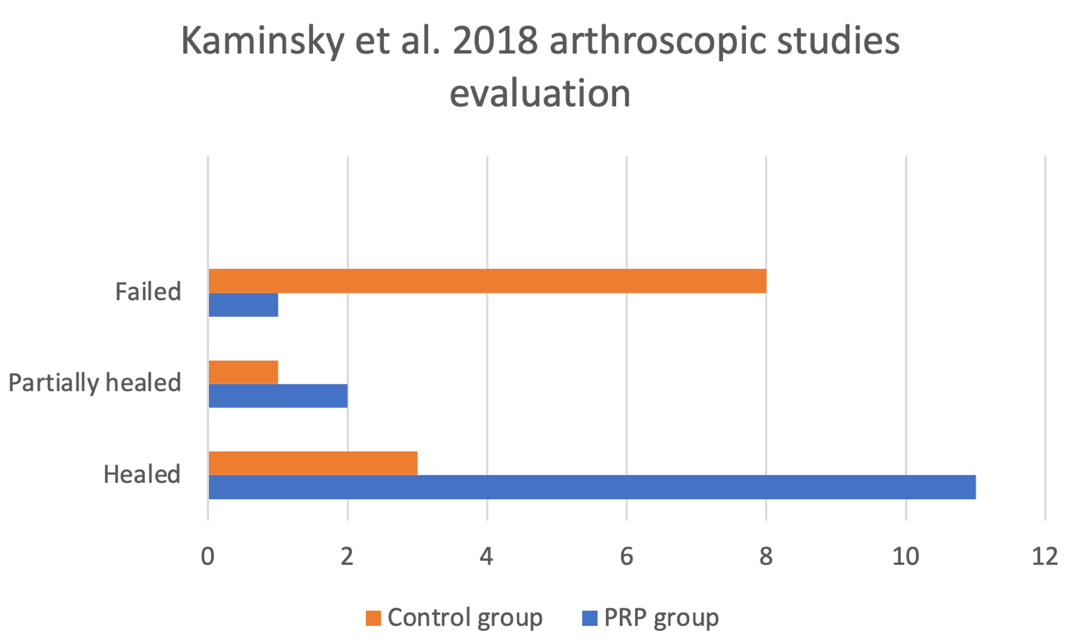


***Supplementary figure number 6:*** Graphic representation of the prevalence of the different meniscal healing grades (previously explained in methods) evaluated cumulatively via MRI + Arthroscopic studies in Kaminsky et al. 2019 [13].


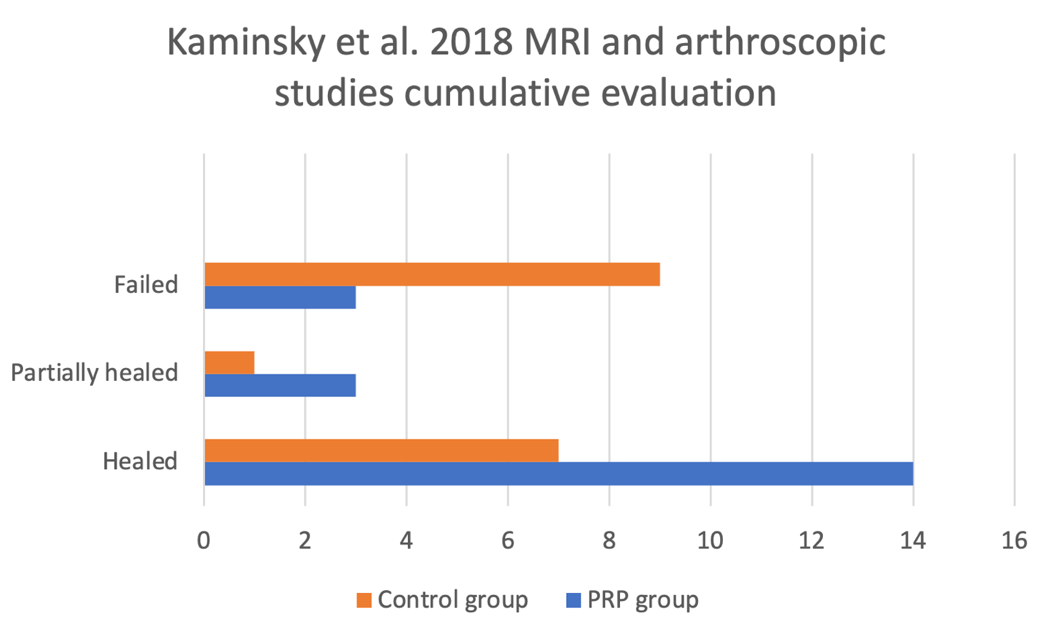


***Supplementary figure number 7:*** Visual representation of the final outcome evaluated trough the several patient-reported tools previously described (KOOS, VAS, IKDC and WOMAC).

A positive sign (green) indicates there was a significative improvement in the PRP-treated group in relation with the indicates clinical evaluation tool compared with the control one, meanwhile the negative sign (red) indicates that there was no significative improvement in the PRP-treated group.


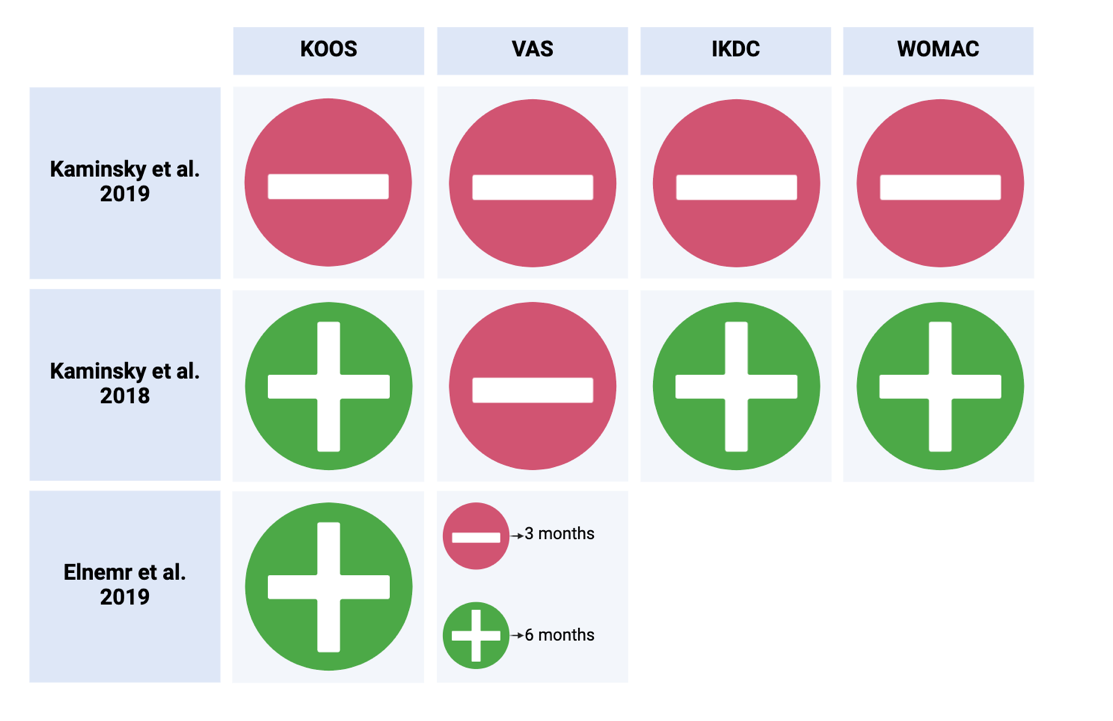


***Supplementary table number 8:*** Possibly bias-leading elements summarized.


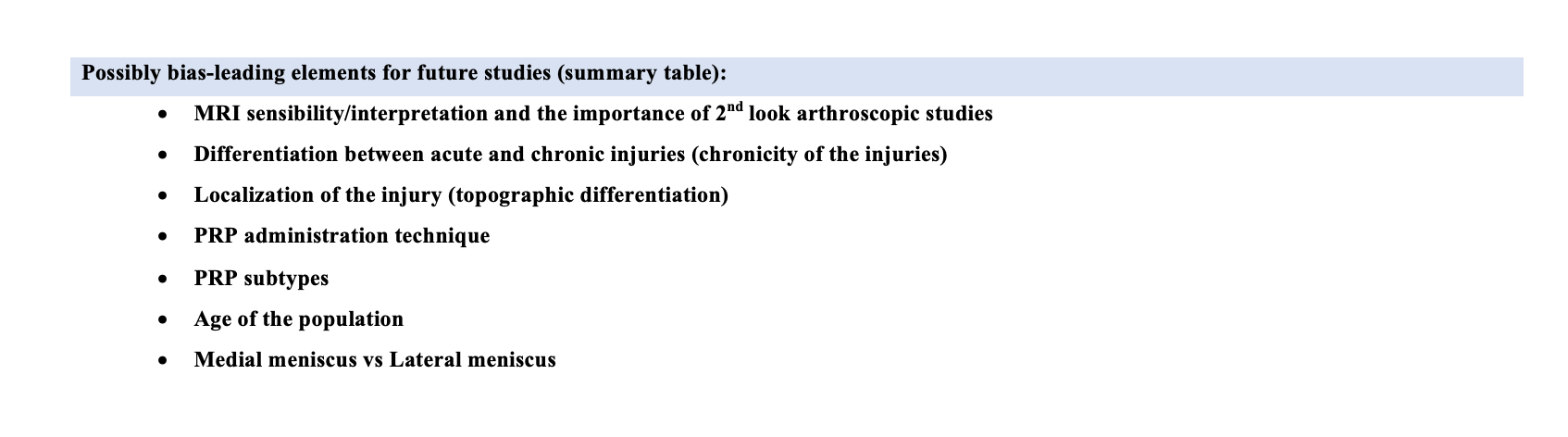


***Supplementary table number 9:*** PICO question of this systematic review.


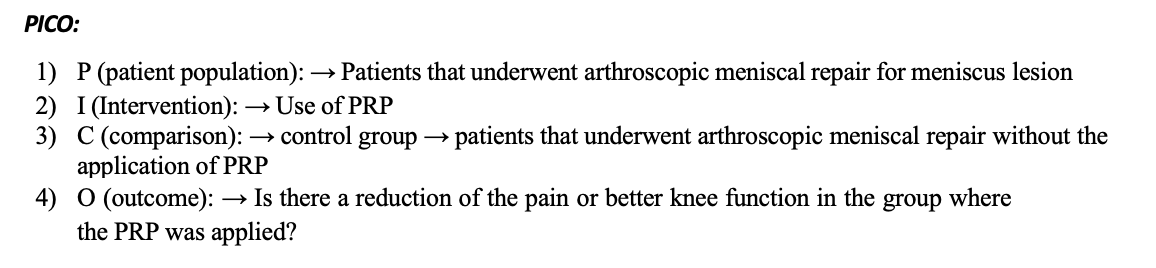

Supplement: Supplementary file 1 — Additional file 1. [file 10195_2024_799_MOESM1_ESM.docx]
